# Study protocol: a randomised controlled trial of the effects of a multi-modal exercise program on cognition and physical functioning in older women

**DOI:** 10.1186/1471-2318-12-60

**Published:** 2012-09-26

**Authors:** Sue Vaughan, Norm Morris, David Shum, Siobhan O’Dwyer, Denise Polit

**Affiliations:** 1 School of Physiotherapy and Exercise Science, Griffith University, Gold Coast, QLD, 4222, Australia; 2 School of Applied Psychology and Behavioural Basis of Health Program, Griffith Health Institute, Griffith University, Gold Coast, QLD, 4222, Australia; 3 Griffith Health Institute, Research Centre for Clinical and Community Practice Innovation, Griffith University, Gold Coast, QLD, 4222, Australia

**Keywords:** Exercise, Cognition, Aged, Multi-modal exercise, Brain derived neurotrophic factor

## Abstract

**Background:**

Intervention studies testing the efficacy of cardiorespiratory exercise have shown some promise in terms of improving cognitive function in later life. Recent developments suggest that a multi-modal exercise intervention that includes motor as well as physical training and requires sustained attention and concentration, may better elicit the actual potency of exercise to enhance cognitive performance. This study will test the effect of a multi-modal exercise program, for older women, on cognitive and physical functioning.

**Methods/design:**

This randomised controlled trial involves community dwelling women, without cognitive impairment, aged 65–75 years. Participants are randomised to exercise intervention or non-exercise control groups, for 16 weeks. The intervention consists of twice weekly, 60 minute, exercise classes incorporating aerobic, strength, balance, flexibility, co-ordination and agility training. Primary outcomes are measures of cognitive function and secondary outcomes include physical functioning and a neurocognitive biomarker (brain derived neurotrophic factor). Measures are taken at baseline and 16 weeks later and qualitative data related to the experience and acceptability of the program are collected from a sub-sample of the intervention group.

**Discussion:**

If this randomised controlled trial demonstrates that multimodal exercise (that includes motor fitness training) can improve cognitive performance in later life, the benefits will be two-fold. First, an inexpensive, effective strategy will have been developed that could ameliorate the increased prevalence of age-related cognitive impairment predicted to accompany population ageing. Second, more robust evidence will have been provided about the mechanisms that link exercise to cognitive improvement allowing future research to be better focused and potentially more productive.

**Trial registration:**

Australian and New Zealand Clinical Trial Registration Number: ANZCTR12612000451808

## Background

Ageing is associated with decline in cognitive functions that are critical to independence, social engagement, and quality of life. Unchecked, this normal and gradual deterioration can advance to clinical cognitive impairment which, in turn, carries a higher risk for progression to dementia [1-3]. As the proportion of people aged over 65 years continues to expand, it is estimated that, by 2050, dementia could affect some 106.2 million people globally [4]. Age-related cognitive decline affects far more people than dementia [5]; so, there is much to be gained at the economic, social and individual levels if an effective, inexpensive approach to age-related cognitive impairment, can be found.

Exercise training has been identified as a promising countermeasure, to age-related cognitive decline [6]. The search for definitive evidence has seen a trend away from non-experimental and quasi-experimental research toward randomised controlled trials (RCTs). Systematic reviews of this literature suggest that formal exercise training resulting in increased cardiorespiratory fitness can improve cognitive performance in older people [7-9]. However, there is still much to be clarified. While cardiovascular exercise demonstrates a moderate effect on cognition, according to a Cochrane review, there is insufficient evidence that cognitive improvements can be attributed to improved cardiovascular fitness [7]. The suggestion is that other factors may be involved and more recently a number of areas have been identified as important targets for future research. These include the motor aspect of exercise [10,11]; and combined modality exercise interventions to address the limitations of repeatedly evaluating a single component of exercise training in relation to cognition [12]. Accordingly this study will adopt a novel approach by combining key emerging themes from the extant literature [13]. Specifically, this study is a RCT of a multi-modal exercise intervention, based on a broad definition of fitness which includes motor (balance, co-ordination, agility, proprioception, flexibility and reaction time) [12] and physical (cardiovascular and resistance) components.

The mechanisms that underpin exercise-induced cognitive gains are also receiving greater attention. This focus has largely been propelled by evidence that indicates that the brain retains a life-long capacity to change and adapt in response to environmental and experiential stimuli, including exercise training [14]. This capacity, referred to as neuroplasticity, has been demonstrated in exercise trained ageing animals [15-18]; and the prospect of similar possibilities in older humans has attracted significant interest [10,19]. As yet, the mechanisms of human brain plasticity (changes in brain structure and function) and cognitive plasticity (changes in cognitive performance), are poorly understood. There are, however, theoretical indications that these changes may be more likely to occur as a result of interventions that involve novelty and complexity that necessitates mental effort [12,15]; otherwise known as cognitive load [20]. Significantly sustained mental effort is a feature of motor skills acquisition. In addition, there is some evidence, mostly from animal studies, that exercise-induced brain changes may be mediated by neurotrophins (proteins that play a role in neurone development), such as, brain-derived neurotrophic factor (BDNF) [19]. BDNF may play a key role in brain plasticity by regulating the growth, maintenance and survival of neurons in the adult brain. Mixed results from the few human studies indicate that poorly understood gender effects may obscure the interpretation of BDNF levels as a biomarker of cognitive function [21]. Recently, Coelho et al. [22] found higher levels of plasma BDNF in response to an exercise intervention in older women (before 351 ± 68 pg/ml and after 593 ± 79 pg/ml; p < 0.001). Accordingly this study will focus on older women.

A much less emphasised but fundamental subject in the extant literature is the question of the relevance of the tasks involved in traditional neurocognitive testing. It is unclear how well performance in various neurocognitive tests translates into the functional abilities and mental processes required for competency in the instrumental activities of daily living for older adults. Furthermore, the extent to which exercise-induced cognitive change correlates with (and mitigates) the processes that characterise age-related cognitive decline, is far from established. The notion of ecological validity or the extent to which test outcomes predict real-life behaviour [23], is not reflected in the present literature, but is increasingly being considered in RCTs generally, by the inclusion of a qualitative evaluation aimed at clarifying the everyday relevance and acceptability of study interventions and their intended or unintended outcomes.

A combination or multi-modal exercise intervention that includes motor as well as physical training and requires sustained attention and concentration, may better elicit the actual potency of exercise to enhance cognitive performance, in later life. Conducted as a randomised controlled trial, the study being undertaken will possess the design parameters with the greatest capacity to test the efficacy of such an intervention. Supplementary qualitative data provides greater insight into the clinical, functional and ecological (every-day) relevance of the intervention. In gathering early data into the effects of exercise on cognitive functioning in women (negating the gender effect) and on BDNF levels (as a biomarker of brain plasticity); more robust evidence will have been provided about the mechanisms that link exercise to cognitive improvement. If these questions can be answered future research may be more precisely focused and potentially more productive.

### Aims of the study

The study focuses on women aged 65–75 years, and aims to:

1. test the effect of a complex, multi-modal exercise program on cognitive functions, physical functioning and a biomarker (BDNF) of brain plasticity;

2. explore the associations between changes in cognitive functions and changes in BDNF, over time and;

3. describe the experience of individuals participating in a multi-modal exercise program.

A multi-modal exercise program has been developed by the first author (SV) in consultation with physiotherapists, exercise physiologists and psychologists. The effects of this exercise program are being evaluated in a randomized controlled trial. The theoretical basis of the program incorporates current understandings derived from the literature on ageing, cognitive neuroscience, psychology and exercise science.

## Methods /design

### Study design

**Figure 1:**
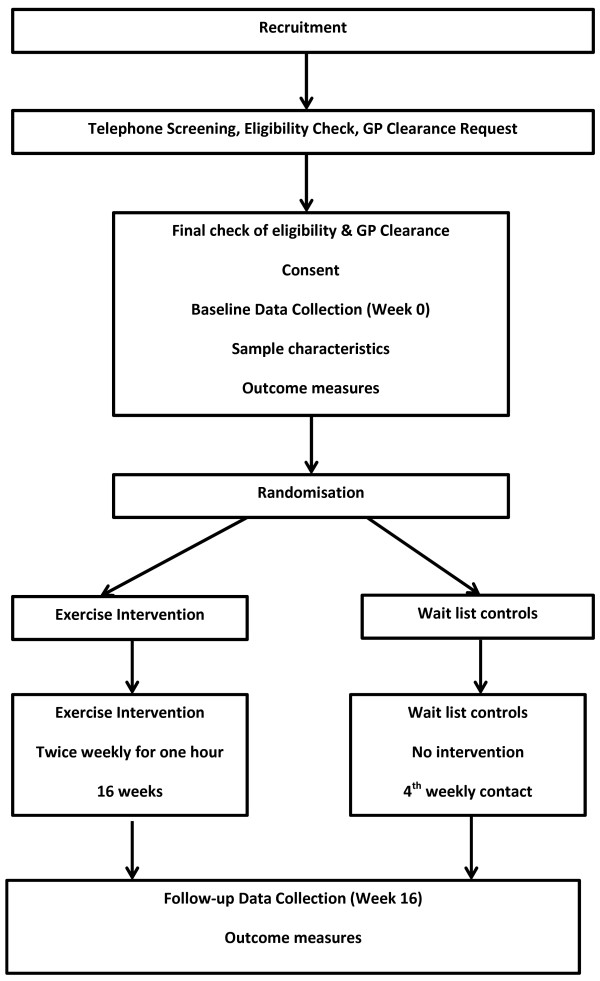
The design of the study.

The study is a 16-week randomised controlled trial of a multi-modal exercise program for women aged 65–75 years. The study is designed to assess the effect of an exercise intervention combining physical fitness training (aerobic and strength) and motor fitness training (balance, coordination, agility, reaction time and flexibility). Participants are randomly allocated to the intervention group or a no exercise wait list control group (See Figure 1). Measurement of primary and secondary outcomes will take place two weeks prior to commencing the exercise training study (baseline) and within one week of completing the 16 weeks exercise or control period. The protocol has been approved by the Griffith University Human Research Ethics Committee (GU Ref No: PES/05/12/HREC).

### Setting

The study is being conducted in community halls on the Gold Coast which is located in South Eastern Queensland, Australia.

### Study population

The program is designed for non-cognitively impaired, community dwelling older adults. Volunteers are recruited through media coverage, local organisations such as ‘Probus’ and ‘Senior Citizens’ and Government agencies such as the Gold Coast City Council. To be considered for the study, individuals must be:

1. female and aged between 65 and 75 years;

2. doing less than 60 minutes of formal exercise training each week; and

3. able to attend classes twice each week on weekday mornings.

### Eligibility criteria

Exclusion criteria include: a score below 31 on the Telephone Interview of Cognitive Status (TICS) [24]; inability to obtain written clearance from a General Practitioner; a diagnosis of dementia or Parkinson’s disease; the inability to walk 20 meters unaided and head injury within the previous 12 months.

### Informed consent

Informed written consent is obtained before baseline testing takes place.

### Sample size

One hundred participants will be recruited for the study. Sample size is determined using a power calculation to detect between group differences in the primary outcome measures. Previous randomised controlled trials of exercise interventions have shown effect sizes between 0.24 and 1.17, in similar populations, for cognitive performance outcome measures, as a result of aerobic interventions [7]. To obtain 80% statistical power with an α level of 0.05, using a t-test for two independent groups and an effect size of 0.6, the sample size required in each of the two groups is 36 participants (G*Power 3.1) [25,26]. Samples of 50 for both the intervention and control groups are recruited, to allow for attrition.

### Randomisation

An automated randomisation service will allocate participants to one of two groups (intervention group or ‘wait-list’ controls). The randomisation service provided by The Clinical Trials Coordinating Centre (CTCC) (Griffith University, Queensland, Australia) is accessed either by telephone or using web-based technology.

### Blinding

Exercise instructors are aware of the allocation of participants; however, all data collectors are blinded to group allocation. Data analysis is conducted by the first author (SV) on a de-identified database.

### Intervention

#### Exercise training intervention

Participants in this group receive a multi-modal exercise class, twice weekly, for a period of 16-weeks. Classes are conducted for 60 minutes and the overall program is designed to include progressions and variations (See Table 1). Each session includes aerobic (cardiovascular), strength (resistance) and motor fitness (balance, co-ordination, flexibility and agility) training in accordance with the ACSM Guidelines for Exercise Testing and Prescription [27] (See Table 2 for details). The cardiovascular component is set to music and choreographed movements are cued in random rather than serial order.

**Table 1:** Overview of three phases of exercise intervention program

**Component**	**Phase 1**	**Phase 2**	**Phase 3**
	**Time**	**Time**	**Time**
**Group freestyle**			
Aerobics	10 mins	15 mins	15 mins
Agility	-	5 mins	5 mins
**Group and/or circuit**			
Strength	15 mins	10 mins	10 mins
Balance	10 mins	10 mins	7 mins
Co-ordination/ Agility/ Reaction time	15 mins	10 mins	7 mins
Cardio	-	-	6 mins
**Group**			
Flexibility and warm down	10 mins	10 mins	10 mins

**Table 2:** Specifics of exercise intervention

**Modality (Type)**	**Format**	**Exercises**	**Examples of exercises**	**Intensity**	**Progressions**	**Variations**
**Phase 1 – Weeks 1-4 – neural adaptation and coordination**						
Cardio (aerobic)	Freestyle aerobics set to music	Antero-postero movements	Marching on the spot	3-4/10 RPE	Increasing number of simultaneous limb movements	Remove lateral movements and shoulder ROM as necessary
		Lateral movements	Three steps forward and back	Music at 124-126 bpm		
		Minimal arm movement				
		Isodirectional upper and lower limb movements	Side step			
			Side tap			
Strength	Group	All major upper, lower body and core muscle groups	Half squats	x2 sets 6-8 reps with light dumbbell (d/b) weights	Commence with no weight progress to light weights (1kg)	Remove weights
			Arm and leg curls			Substitute wrist or ankle weights as necessary.
			Seated theraband rows			
Balance	Group	Static balance with decreasing support as able	Supported standing on one leg	N/A	Increased time + Challenges to concentration	One-to-one supervision and support as necessary
Coordination Agility	Some elements included in free style aerobics	Manoeuvring around and stepping over objects	Weaving in and out of chairs	N/A	Chairs closer	One-to-one supervision and support as necessary
Reaction time	Group	Flat foot heel drumming	Fast, fixed-pattern, foot tapping (seated)			
			Wall ball throws			
Flexibility + warm down	Group	Static stretches on floor	Cat and camel stretches	3-4/10 RPE Minimum 20 secs	Vary with musculo-skeletal status	Substitute as necessary
			Lying hamstring stretches			
			Back extensions			
**Phase 2 – Weeks 5-11 – conditioning**						
Cardio (aerobic)	Freestyle aerobics set to music	Antero-postero movements	As for Phase 1 plus	4-5/10 RPE and 126-128 bpm music		Participant sets own pace as able
		Lateral movements	Zig zag movements			
		Multiple direction changes	Knee lifts			
			Lateral lower body with			
		Increased strength component	antero-postero upper body			
		Arm movement	movements			
		Non-isodirectional upper and lower limb movements				
Strength	Circuit	All major muscle groups	Different stations	Recommended RPE = 4-5/10 2x 30 second intervals per station	Increase weights as able	Remove/reduce weights
		Compounded exercise	including:			
			Continuous rolling movement on mat			Substitute wrist or ankle weights as necessary.
			Fitball squats			
			Weighted bag drags			
			Ball bouncing			
Balance	Circuit	Static and dynamic balance	Heel-toe (walk the line) One-leg pose with ball throws	2x 30 second intervals per station	Challenges to concentration	One-to-one supervision or remove element (e.g. ball throws)
Coordination Agility	Free style aerobics + Circuit + Group	Circuit:	Circuit:	Circuit:	Faster movements as able	One-to-one supervision and reduce speed of movement
Reaction time		Dynamic combination/compounded exercises	Flat foot heel drumming	2x 30 second intervals per station	Substitute smaller balls	
		Group:	Walking ball bounces		Alternate between dominate & non-dominate hands	
		Dynamic knee lifts alternating with rapid foot movements	Group:			
			Various moving foot sequences			
			Catch dropping objects (noodle drops)			
Flexibility + warm down	Circuit + Group	Circuit: Spinal rotation	Circuit: Alternating wall taps	Circuit: 2x 30 second intervals	Increase according to tolerance & musculo-skeletal status	Decrease with musculo-skeletal status
		Group: Static stretches on floor	Group: Cat and camel stretches	Group: 3-4/10 RPE Minimum 20 s		
			Lying hamstring stretches			
			Back extensions			
**Phase 3 – Weeks 12-16 – conditioning**						
Cardio (aerobic)	Freestyle aerobics set to music	Antero-postero movements	As for phases 1 and 2 plus:	5-6/10 RPE and 126-128 bpm music	Participant sets own pace as able (increases as able)	Participant sets own pace (decreases intensity or complexity of movement as required)
		Lateral movements	‘Pride of Erin’ dance steps			
		Multiple direction changes	Marching with alternating parallel and 45 degree angle arm movements			
		Increased demands on postural control				
		Higher arm movement				
		Non-isodirectional upper and lower limb movements				
Strength	Circuit	All major muscle groups	Lunges	Recommended RPE = 5-6/10 2x 40 second intervals per station	Increase weight as able	Remove/reduce weight or substitute exercises targeting same muscle group as tolerated
		Compounded exercise	D/b flyes supine on roller Modified (wall) push ups			
Balance	Circuit	Static and dynamic balance	Stand on foam	2x 40 second intervals per station	Introduce perturbation/reduce base of support	One-to-one supervision or remove element (e.g. ball throws)
			Step onto foam and assume one leg pose			
			One legged stand and reach			
Coordination Agility	Free style aerobics + Circuit + Group	Circuit:	Circuit:	Circuit:	Faster movements as able	One-to-one supervision and reduce speed of movement
Reaction time		Dynamic combination exercises	Noodle drops	2x 40 second intervals per station		
		Group:	Group:			
		Dynamic knee lifts in combination with rapid foot movements	Freestyle aerobic routine + noodle			
Flexibility + warm down	Circuit + Group	Circuit: Spinal rotation	Circuit: Alternating wall taps	Circuit:	Increase with musculo-skeletal status	Decrease with musculo-skeletal status
		Group: Static stretches on floor	Group: Cat and camel stretches	2x 40 second intervals per station		
			Lying hamstring stretches			
			Back extensions			
			Quadruplex			

Strength training incorporates progressive weight training and weight bearing exercises involving the major muscle groups. Motor training involves both static and dynamic balance, coordination and agility requiring manoeuvring around and stepping over objects. Reaction time training includes ball activities and flexibility training involves dynamic and static stretches for all major muscle groups (See Table 2). Sessions are conducted by accredited fitness instructors, with a maximum of 20 participants and an instructor-to-participant ratio of 1:10.

### Control group

The control group is on a waiting list to attend the 16-week exercise program which commences at the end of the study; and after the post intervention measures have been completed by both groups. The classes are offered free of charge. Control group participants are contacted by telephone every 4 weeks to foster an ongoing sense of engagement in and relevance to the study.

### Intervention fidelity

Two instructors are present in each class. Only these two instructors will administer the intervention. At random intervals an independent assessor will observe all classes and monitor for content consistency using a check-list based on the explicit components of the exercise intervention protocol. In addition, instructors will maintain a log of activities in each class and these logs will also be reviewed by the independent assessor to check for intervention fidelity.

### Adherence to intervention protocol

Trained fitness instructors document attendance at each class. Intervention group are asked to plan to attend at least 85% of classes and are followed up by telephone if they are absent for two consecutive classes. In order to maximise participant contact and follow-up participants in both groups are asked to provide at least two sets of contact details; direct and via a friend or family member.

### Assessment protocol

#### Screening

Information sessions about the study are conducted at a number of local organisations that are attended by older adults. Individuals who express interest in the study at the information sessions are asked for their contact details and initial checks of age group and the ability to walk 20 meters unaided are completed. Individuals with preliminary eligibility are issued with General Medical Practitioner (GP) clearance forms which are subsequently used should the screening telephone interview reveal the requirement for GP clearance.

The formal screening process is conducted by telephone and takes approximately 15 minutes. It includes the TICS and the Pre-Activity Readiness Questionnaire (PAR-Q)[27]. Individuals who are otherwise eligible and who report a serious medical condition are asked to take clearance forms to their GPs to obtain written permission to participate. Once individuals have been assessed as being eligible to participate they are invited to attend a data collection and randomisation session where demographic and health information is collected along with baseline outcome measures.

### Baseline demographics, current activity levels and health information

Details of age, education, marital status, occupation and language used at home are collected by interview. Participants are asked to give details of any medical conditions that may affect physical activity (using the PAR-Q). They are asked to complete the 21 item Depression, Anxiety and Stress Scale (DASS-21) which has good internal consistency as well as convergent and discriminant validity, especially for the depression scale [28]. In addition, all participants will be fitted with a pedometer for five days to record activity levels at baseline.

### Outcome measurement

The primary outcome measure is neurocognitive test performance related to the processes of working memory, inhibition, shifting, verbal fluency and simple and complex reaction time i.e. executive function. These functions are highly associated with age-related cognitive decline, mobility and the ability to perform activities of daily living [29-31]. A recent randomised controlled trial of a resistance training program used similar primary outcome measures and demonstrated an effect for a twice a week exercise program [32]. The secondary outcome measures are blood serum levels of BDNF, physical and functional capacity including the 6 minute walk test, the ‘timed up and go’ test and the one-leg stance test of balance; and anthropometric values related to girth circumference at the waist and hip, resting heart rate and blood pressure.

Physical functioning and cognitive assessment are conducted in the community (in halls) by exercise physiologists and psychologists, respectively. Anthropometric measurements are completed by a registered nurse. Cognitive testing requires less than 60 minutes and precedes physical assessment which lasts for 30–40 minutes. Participants are transported to an accredited blood collection facility. Blood samples are then conveyed to a laboratory for assay of plasma Brain Derived Neurotrophic Factor (BDNF).

### Primary outcome measures

#### Neurocognitive Tests

##### California older adult stroop test (inhibition)

Stroop tests assess the ability to suppress a habitual response in favour of an unusual response [33,34]. They generally demonstrate good test-retest reliability (0.90, 0.83 and 0.91 for the three parts of the test) [35] and minimal susceptibility to practice effects [36]. The COAST is a Stroop test developed specifically for use with older populations [37]. Participants are required to first name colours (Colour), then read colour name words (Word); and then name ink colours when the names of colours are printed in a different (non-corresponding/incongruent) colour ink. During the incongruent condition, the two conflicting sources of colour information cause a competing effect (Interference) which is most typically observed as a prolonged reaction time compared to the neutral or congruent conditions [38].

##### Controlled oral word association test (COWAT) (verbal fluency)

Different forms of the COWAT test measure verbal fluency and also draw on executive function and memory. Participants are given one minute to generate as many words as possible that begin with a specific letter. This task is repeated three times with three different letters (e.g. F, A, S). These tests generally demonstrate inter-scorer reliability of 0.70, retest reliability of 0.88 and concurrent validity [39].

##### Letter-number sequencing test (LNS) (working memory)

The Letter-Number Sequencing test [40] measures working memory as well as sequencing, attention and concentration abilities. The participant is read a combination of numbers and letters and is asked to recall the numbers first in ascending order and then the letters in alphabetical order. Each item consists of two trials, and each trial is a different combination of numbers and letters. There are seven items ranging from 2-letter/number sequences (e.g., B-7) to 8-letter/number sequences (e.g., S-2-L-8-B-1-G-7). The maximum score possible is 24 points. The validity and reliability of this test is well established in older adults with test-retest reliability in the range of 0.70 to 0.80 and a factor loading of 0.62 with the Working Memory Index [41].

##### Psychomotor speed (simple reaction time and complex reaction time)

Reaction Time, the time between presentation of a stimulus and initiation of a response, is said to reflect psychomotor and processing speed; and is assessed with 1-choice and 4-choice stimulus conditions. Using computer based testing the first stimulus condition requires identification of a single object (e.g., yellow circle). The second stimulus condition requires the discrimination of a single object, amongst 4 object choices. Simple reaction time (SRT) and 4-choice reaction time (CRT) are calculated as the time (milliseconds) from perception (release of press-pad) to touch of appropriate stimulus on the screen. Split half reliability of up to 0.90 has been reported for an 18 task trial [35]

##### Trail making test (TMT) (part A & B) (executive function and shifting)

The TMT is considered to be a test of visual search, attention, flexibility of thinking, motor function, and executive function [34,36,39]. Part B of the TMT requires the individual to mentally shift between two well-rehearsed sequences (numbers and letters) as quickly and as accurately as possible. Shifting ability represents the capacity to adapt to changes in the environment by switching from one mental set to another.

In Part A, the task is to connect the numbers in ascending order (i.e.1-2-3-4…). Part B involves alternately connecting numbers and letters in ascending order (for the numbers) and sequential order for the alphabet letters (i.e. 1-A-2-B…). The score derived for each trail is the number of seconds required to complete the task [39]. Reliability tests for the TMT range from 0.60 to 0.90 [41] and the test is particularly useful for detecting early stages of dementia [41].

### Secondary outcome measures

#### Brain derived Neurotrophic factor (BDNF)

Brain derived neurotrophic factor (BDNF) is a protein and the most abundant neurotrophin in the brain, with an important role in brain neurogenesis, synaptic plasticity, learning and memory [42,43]. The brain has been found to be the main source of BDNF concentrations in venous blood (circulating BDNF) [44]. Participants have their usual breakfast (1–2 hours before venipuncture) and are asked to refrain from exercise for 36 hours prior to blood draw. Eight milliliters of venous blood is drawn into EDTA tubes in the morning, to control for diurnal range variation in plasma BDNF levels [45]. The samples are processed according to the manufacturer’s specifications. Plasma is obtained by centrifugation of blood tubes for 15 min at 2000 × g and 24°C and is aliquoted and stored at −80°C until measured. Plasma samples are assessed for BDNF concentrations using a commonly used [45] commercially available two-site sandwich enzyme-linked immunoabsorbent assay (ELISA) kit (R&D Systems, Minneapolis, MN, USA).

#### Physical functioning: Six minute walk test (6MWT)

The Six-minute walk test is a functional validated measure of aerobic fitness (exercise capacity); and is based on the number of meters walked in six minutes [46]. The 6MWT is performed indoors, along a 30 meter walking course that is flat and straight with a starting line and turnaround point clearly marked. The total distance walked is tallied using pre-marked intervals as a guide. The 6MWT is reported to have strong test-retest reliability when used before and after cardiac rehabilitation programs [47]. A recent study has confirmed that while there is a learning effect that can increase the distance walked; when used as an outcome measure, one measurement is sufficient to show change in performance over time [47].

#### Physical functioning: Timed Up-and-Go

This is a clinical performance based measure of mobility, lower extremities function and fall risk. It is normally distributed, related to executive function and suitable for the assessment of healthy older adults [48]. The TUG test (TUGT) will be conducted using a chair with arms and a seat height of 46 cm placed upon a flat, surface with cones marking the 3 m turning point [49]. Subjects are instructed: “On the word ‘go’, get up and walk as quickly and as safely as possible to cross the line marked on the path, turn around, walk back and sit down”[49]. The activity will be timed from the subject’s back leaving the back of the chair to the return of the subject to this same position. Using a standardised protocol, each subject will be required to perform one untimed, practice TUGT followed by two timed TUGTs (i.e. TUGT1 and TUGT2). At least four minutes of seated rest occurs between each TUGT.

#### Physical functioning: balance test (the one-leg stance test)

The one-leg stance test [50] requires participants to stand unassisted on one leg with hands on hips. Participants are asked to stand on one preferred leg, flex the opposite knee allowing the foot to clear the floor; then balance on the one leg for as long as possible. Timing begins when the leg is lifted and is timed in seconds until the person returns the non-weight bearing foot to the ground. The test has good reliability in older adults (ICC =0.92) [51].

#### Anthropometric measures

Weight is measured using a digital flat weighing scale with a non-slip glass platform. Height (using a stadiometer), waist circumference and hip circumference are measured in centimeters. The waist is measured at the smallest circumference around the torso between the end of the xiphisternum and the top of the iliac crests. The hips are measured at the greatest circumference between the iliac crests and the upper femur. Resting blood pressure is measured using a sphygmomanometer (Welch Allyn 767 Series mobile aneroid sphygmomanometer) and stethoscope. Resting heart rate is calculated by palpation of the radial pulse for one minute. The anthropometric measurements are conducted by a registered nurse.

### Analyses

The data analysis for the randomised controlled trial will be undertaken on an intention-to-treat basis. To characterize the sample in this study, means or proportions and standard deviations (SDs) will be calculated by randomisation group for multiple baseline demographic, cognitive functioning and anthropometric variables. Unadjusted means and SDs for each cognitive performance measure obtained at the baseline and follow-up visits will be calculated. Missing outcome data will be imputed using multiple imputation [52]. Analysis of covariance will be undertaken for 16 week follow-up outcome measure scores, controlling for baseline cognition (TICS), and using baseline outcome values as covariates. Ninety- five per cent confidence intervals and effect sizes (standardised mean differences) will be calculated for each outcome measure. Differences in follow-up secondary outcome measure scores will be assessed for normality of distribution and either parametric or non-parametric statistical analyses applied as appropriate (e.g. ANCOVA, logistic regression etc.). Hierarchical multiple regression analyses will be used to investigate associations between plasma BDNF levels and a range of independent variables including demographic, anthropometric, cognitive functioning and physical functioning variables.

### Qualitative component

A qualitative component is embedded in the study to elicit information about the participants’ experience of the intervention and perception of changes to physical and cognitive function. This component of the study will employ a triangulation approach to the incorporation of qualitative and quantitative data at the stage of results interpretation [53].

Face to face, audiotaped, individual interviews are undertaken with a sub-set of the study population allocated to the intervention group. Interviews are conducted with 12 to 15 participants who give written informed consent. The final number of interviews will be determined by data saturation which will be revealed during concurrent analysis. The interviews commence with questions such as, “Please describe a typical exercise class”, “What aspects of the class did you find most/least enjoyable?” “What aspects of the class did you find least/most beneficial?” “Why?” “What changes if any, have you noticed about yourself that you think may relate to class attendance?” Verbatim transcription of data will be undertaken and thematic analytic techniques will be used to analyse the data [54].

## Discussion

A future study, involving multi-modal exercise training and conducted in the form of a randomised controlled trial would be expected to extend previous work in a number of significant ways. Firstly, an adequately powered randomised controlled trial would fulfil a methodological recommendation that has consistently been made in systematic reviews of the extant literature. Secondly, to our knowledge, there are currently no studies where this particular design has been used to test whether the combination of cardiovascular, strength and motor fitness training can elicit cognitive effects in later life. Thirdly, a theory-driven intervention design allows a targeted approach that increases the likelihood of eliciting improved cognitive performance in the very regions of the brain that have most pertinence to age–related decline. Adding motor exercise training to an exercise intervention deliberately introduces the ingredient of movement based novelty and complexity [12,15]. Motor training requires sustained concentration and attention and is therefore inherently cognitively demanding [55]. Increased cognitive load engages higher order cognitive processes that largely activate the prefrontal cortex and it is this area of the brain that is said to most evidence the early declines associated with the ageing process [42]. Fourthly, while a properly designed randomised controlled trial is the gold standard for assessing the efficacy of an intervention in terms of statistical significance [56], the real-life relevance of outcomes may be better reflected in terms of the clinical significance of the study results. To this end, this study will triangulate psychometric data with qualitative data, to better reflect the impact of the intervention on the everyday experience of older women. Finally, there is still a gap in the literature regarding the mechanisms that might underpin the cognitive effects of exercise. Animal models have generated great interest in the role of BDNF and human studies demonstrate promise, however, more evidence is needed. Together the particular combination of design characteristics proposed for this study is expected to make a unique contribution to the current body of knowledge by furthering our understanding of the relationships between multi-modal exercise, age, cognitive function and BDNF in older women.

## Competing interests

The authors declare that they have no competing interests.

## Authors’ contributions

All authors contributed to the design of the study. SV drafted the manuscript, while NM,DS SO and DP contributed to drafts of the manuscript. All authors have read and approve the publication of the final manuscript.

## Pre-publication history

The pre-publication history for this paper can be accessed here:

http://www.biomedcentral.com/1471-2318/12/60/prepub
